# Osteolytic Lesions (Brown Tumors) of Primary Hyperparathyroidism: A Report of Two Cases

**DOI:** 10.7759/cureus.61708

**Published:** 2024-06-05

**Authors:** Abrar M Alrotoie, Asia A Aljohani, Renad Alrehaili, Mayar Alharbi, Yousef M Alalawi

**Affiliations:** 1 Medical School, Taibah University, Medina, SAU; 2 Medicine and Surgery, Al Rayan College, Medina, SAU; 3 Endocrinology, Diabetes and Metabolism, Prince Mohammed Bin Abdulaziz Hospital, Medina, SAU

**Keywords:** tumors, endocrine, giant cells, primary hyperparathyroidism, brown tumors

## Abstract

Primary hyperparathyroidism is characterized by excessive production of parathyroid hormone. As the condition progresses, bone loss primarily occurs due to resorption. A complication of this condition is the formation of fibrotic and cystic changes in the bone, known as brown tumors. These lesions occur in areas of significant bone resorption, where fibrovascular tissue and giant cells replace bone tissue, often accompanied by hemorrhage and hemosiderin deposits. These brown lesions are rare, with an occurrence rate ranging from 1.5% to 4.5%. We present two cases of middle-aged women who had presentations consistent with hyperparathyroidism and presented with complications such as bone pain and numbness. Both underwent parathyroidectomy to manage the cause and recovered after the surgery. These cases emphasize the importance of recognizing primary hyperparathyroidism as a potential cause of abnormal lesions and highlight the diverse presentations associated with this condition.

## Introduction

Brown tumors and parathyroid adenomas are distinctive but interconnected conditions often encountered in cases of primary hyperparathyroidism (PHPT). This systemic disorder is characterized by excess parathyroid hormone (PTH) secretion and resultant hypercalcemia. Among the various manifestations of PHPT, renal calculi, brown tumors, and parathyroid adenomas pose significant clinical challenges due to their potential complications and influence on patient care [1]. Parathyroid adenomas are considered to be the main etiology in about 85% of PHPT cases [2].

Brown tumors, also known as osteoclastomas or osteitis fibrosa cystica, are non-cancerous bone lesions arising from prolonged hyperparathyroidism [2]. These lesions stem from increased osteoclastic activity and are characterized by fibroblastic proliferation, hemorrhage, and hemosiderin deposition within bone tissue. Though once considered rare, improved recognition and diagnostic methods have led to an increased detection rate. Brown tumors can affect various bones, typically the long bones, pelvis, ribs, and facial bones. They may clinically present with bone pain, deformities, fractures, or palpable masses, often mimicking malignant or benign bone lesions [3].

In contrast to brown tumors, parathyroid adenomas are benign tumors of the parathyroid glands and represent the most common cause of PHPT [2]. These adenomas autonomously secrete PTH, disrupting calcium balance. While the exact cause remains unclear, factors such as genetics, environment, and hormones have been implicated [4]. Patients with parathyroid adenomas commonly exhibit symptoms related to hypercalcemia, including fatigue, weakness, kidney stones, and neuropsychiatric issues. Additionally, parathyroid adenomas may increase the risk of complications such as osteoporosis, kidney dysfunction, and heart disease [1]. In this article, we present the radiological and clinical features of two cases of brown tumors caused by parathyroid adenomas.

## Case presentation

Case 1

A 66-year-old Saudi woman presented to the clinic complaining of general weakness, fatigue, numbness in her hands, and bone and muscle pain for more than one year. She had a history of type 2 diabetes mellitus, dyslipidemia, hypertension, and stage 3 chronic kidney disease. Her baseline creatinine was 90 µmol/L. She had no history of previous fractures, constipation, or urinary symptoms. Her physical examination was unremarkable, except for lumbar spine tenderness. Subsequent blood tests demonstrated PTH at 958.8 pg/mL (normal range: 16-67 pg/mL), phosphate at 0.52 mmol/L (normal range: 0.75-1.51 mmol/L), adjusted calcium at 3.1 mmol/L (normal range: 2.21-2.4 mmol/L), and alkaline phosphatase at 169 (normal range: 41-149 U/L). A thyroid ultrasound showed a heterogeneous area (2 × 1.5 cm) right posterolateral to the thyroid gland and posterior to the right common carotid artery, appearing separate from it. This area showed irregular chunky calcifications and no vascularity on Doppler evaluation. There were no pathologically enlarged cervical lymph nodes.

A diagnosis of PHPT was made. She underwent left inferior and right inferior parathyroidectomy. The abnormal parathyroid gland was excised. Parathyroid adenoma was diagnosed based on intraoperative frozen section pathological examination and confirmed by postoperative pathology. Her postoperative laboratory results showed PTH at 1,452.5 pg/mL and adjusted calcium at 3.03 mmol/L. She was given calcium carbonate and alfacalcidol for possible postoperative hypocalcemia. The patient was discharged and planned to follow up with endocrinology regarding her persistent elevated PTH level.

**Figure 1:**
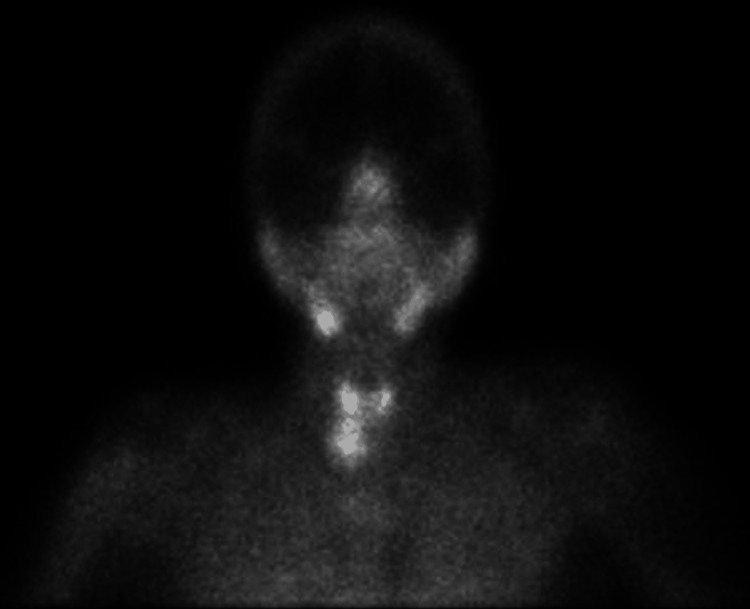
A parathyroid technetium 99m sestamibi scintiscan showing abnormally high uptake observed at the lower pole of the right thyroid lobe.

**Figure 2:**
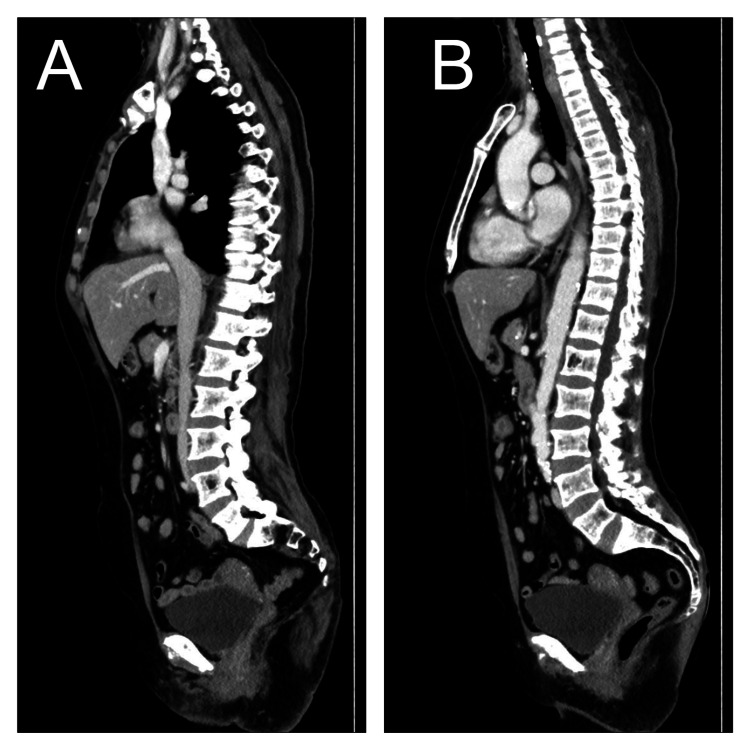
Abdominopelvic computed tomography imaging shows generalized marked osteopenia with small benign-looking osteolytic lesions involving lumbar vertebral bodies.

The patient was called from home for a critical lab result: her adjusted calcium level was 3.61 mmol/L. In the emergency department, her adjusted calcium was 3.5 mmol/L, and she complained of generalized bone pain. She was managed by intravenous fluid, zoledronic acid, and calcitonin. Further investigation was performed to look for possible malignancy. Computed tomography (CT) of the neck showed an oblong, slightly lobulated soft tissue mass (measuring 3.4 × 1.9 × 2.3 cm) that was posteroinferior to and separate from the right lobe of the thyroid gland. It showed internal cystic areas with curvilinear calcifications. PTH technetium 99m sestamibi scintigraphy showed discordant focal increased uptake observed inferior to the right thyroid lobe measuring around 2.5 × 2 cm (Figure 1). CT of the abdomen and pelvis revealed generalized marked osteopenia with small benign-looking lytic lesions involving the L1, L3, and L4 vertebral bodies, likely brown tumors (Figure 2).

She underwent a right inferior parathyroidectomy. Her PTH dropped from 1,218.2 pg/mL before excision to 187.40 pg/mL 20 minutes after removal. Neoplastic parathyroid with vascular and capsular invasion was diagnosed by intraoperative frozen section. The patient recovered well and was discharged on vitamin D (2,000 IU daily), with a follow-up appointment with endocrinology.

Case 2

**Figure 3:**
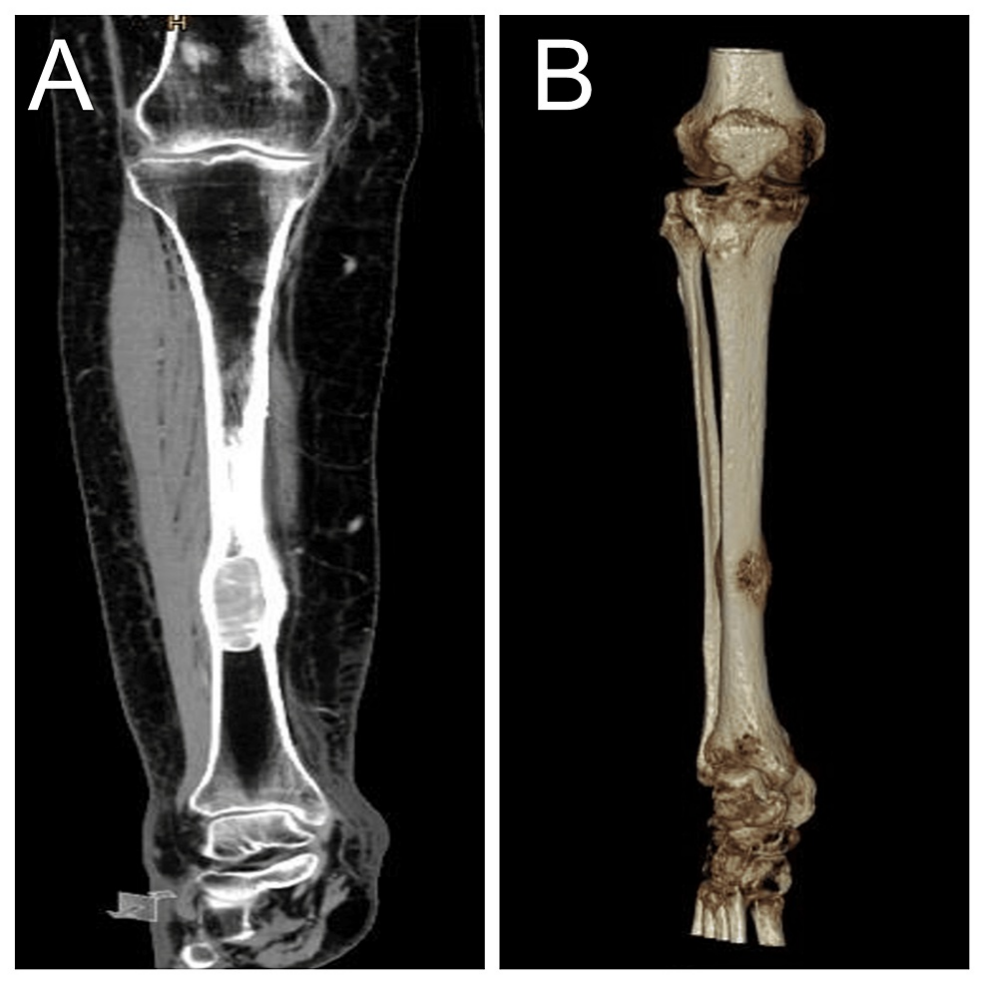
Tibia/fibula computed tomography (A) bone window (B) three-dimensional view showing a well-defined, slightly expansile, oval-shaped, intramedullary osteolytic lesion in the distal third of the diaphysis of the right tibia.

A 50-year-old Saudi woman with no medical history presented to the clinic complaining of chronic right leg pain and was using a wheelchair due to severe pain. She denied a history of trauma, fragility fractures, kidney stones, or polyurea. A lower limb CT scan showed an oval-shaped intramedullary lytic lesion (5 × 2.6 × 2.2 cm) in the distal third of the diaphysis of the right tibia (Figure 3). It had caused endosteal cortical scalloping and some cortical breaks/destruction associated with a minimal periosteal reaction. Lumbar/thoracic X-ray showed abnormal bone texture and heterogeneous trabeculae. These findings were suggestive of brown tumor lesions. Laboratory tests showed elevated serum levels of PTH (498 pg/mL), adjusted calcium (2.60 mmol/L), and alkaline phosphatase (330 U/L), and a low phosphorus serum level (0.45 mmol/L). Her vitamin D level (55.8 nmol/L; normal range: 50-125 nmol/L), albumin, estimated glomerular filtration rate (eGFR), and creatinine were within normal limits. A thyroid scan revealed a single left parathyroid adenoma.

A diagnosis of PHPT with brown lesions was made, and she underwent left inferior parathyroidectomy. Parathyroid adenoma was diagnosed based on intraoperative frozen section pathological examination and confirmed by postoperative pathology. The patient was discharged and given vitamin D 50,000 IU every two weeks and calcium carbonate 600 mg twice a day.

Six months after her parathyroidectomy, she was referred to the clinic regarding her persistently elevated PTH level. She no longer complained of leg pain and was walking with no assistance. Her serum levels were as follows: PTH at 112.7 pg/mL, adjusted calcium at 2.23 mmol/L, and vitamin D at 79.7 U/L. Her eGFR and creatinine were within normal limits. The patient was non-adherent to her medications. She was told about the importance of vitamin D replacement in her case. She was followed up later in the clinic and her PTH level had normalized.

## Discussion

PHPT manifests as an endocrine disorder characterized by the overproduction and release of PTH from one or more of the four parathyroid glands. The predominant etiology of PHPT is parathyroid adenoma, accounting for roughly 80% of cases, followed by parathyroid gland hyperplasia, which constitutes 15%-20% of cases. Parathyroid carcinoma, albeit rare (occurring in fewer than 1% of cases), can also precipitate PHPT. Conversely, secondary hyperparathyroidism arises as a complication of conditions such as vitamin D deficiency or chronic renal failure [5]. The reported incidence of PHPT is approximately 22 per 100,000 individuals per year. This condition exhibits a female predilection and tends to peak in occurrence during the sixth to seventh decade of life [6].

The rising prevalence of PHPT is attributed to enhanced diagnostic practices and routine monitoring of calcium levels, resulting in a predominance of asymptomatic cases among patients. Conversely, in regions with limited screening protocols, particularly in developing nations, PHPT often presents with pronounced symptomatic manifestations involving the skeletal and renal systems. Notably, skeletal manifestations tend to emerge later in the disease progression and are infrequently documented in the literature. Common skeletal presentations encompass diffuse osteopenia, subperiosteal bone resorption, fractures, and the occurrence of multiple circumscribed lytic lesions, referred to as brown tumors [5].

In our cases, the predominant skeletal manifestation was a brown tumor. These benign osteoclastic lesions are rare, with an occurrence rate ranging from 1.5% to 4.5%, and typically present as a late manifestation of PHPT, with potential involvement of the entire skeletal system. Clinical manifestations of brown tumors may include bone pain, pathological fractures, or asymptomatic presentations. Their appearance as diffuse osteolytic lesions can lead to misinterpretation as metastatic lesions. The pathogenesis of brown tumors involves excessive osteoclastic activity resulting in bone resorption, subsequently replaced by fibrotic tissue and giant cells. The predilection sites for brown tumors commonly include the clavicle, jaw, ribs, and pelvis, with less frequent occurrences noted in the cranial region [7]. Notably, in our cases, the lumbar spine and tibia were the affected sites.

The clinical presentations of these neoplasms exhibit variability and lack specificity, encompassing symptoms such as weakness, weight loss, bone pain, or pathological fractures, occasionally manifesting as progressive bone enlargement or urinary lithiasis [7]. Our patients reported bone pain as the primary complaint. The traditional manifestations of hypercalcemia are encapsulated in the mnemonic “bony pain/bone fractures, renal stones, abdominal groans, and psychic moans.” Additional associated symptoms observed in patients with hypercalcemia include paraesthesia, headaches, recent fractures, constipation, polyuria, and polydipsia. Nevertheless, the majority of patients remain asymptomatic and are typically identified incidentally during routine clinical assessments [2].

From a biological perspective, the diagnosis of PHPT hinges upon the concurrent presence of hypercalcemia and hyperparathyroidism, coupled with hypophosphatemia and hypercalciuria [7]. In cases of suspected or confirmed hyperparathyroidism, cervical ultrasound and technetium 99m sestamibi scintigraphy are frequently used radiological modalities. Ultrasonographic examination typically identifies parathyroid adenomas as well-defined, hypoechoic, homogenous lesions situated in the inferior pole of the thyroid gland. The smaller size of these adenomas may render them imperceptible, necessitating meticulous evaluation of the inferior thyroid pole. With technetium 99m sestamibi scintigraphy, both the thyroid and parathyroid glands initially exhibit radiotracer uptake in early imaging phases. However, while thyroid uptake diminishes in later images, parathyroid uptake persists and intensifies over approximately two hours. This persistent and heightened uptake in parathyroid adenomas distinguishes them from the surrounding thyroid tissue [7].

On imaging, brown tumors typically manifest as lytic bone lesions. They appear circumscribed and can occur as single or multiple entities, with a morphology that may range from simple to polylobular. Occasionally, they exhibit bone expansion and extension into adjacent structures, which can lead to pathological fractures. These lesions may display a mixed appearance, showing characteristics of both sclerotic and lytic lesions. Their contours may be ill-defined, potentially leading to confusion with malignant origins, necessitating histological confirmation. However, distinguishing between a brown tumor and a giant cell tumor through histology can be challenging. Hence, it is crucial to consider the presence of hypercalcemia and hyperparathyroidism for an accurate diagnosis [7].

Histologically, brown tumors are characterized by a vascular fibroblastic stroma and numerous osteoclast-like multinucleated giant cells, often interspersed with hemorrhagic infiltrates and hemosiderin deposits [2]. The primary differential diagnoses include bone metastases, multiple myeloma, sarcomas, giant cell tumors, tonsillar cysts, chondromas, and aneurysmal bone cysts [7].

Regarding treatment, the key approach for PHPT is the surgical removal of the hyperactive parathyroid tissue, as was done in the present cases. Surgery commonly alleviates clinical symptoms, corrects biochemical irregularities, and enhances bone mineral density. Managing bone tumors hinges upon addressing their underlying cause. However, in specific cases, lesion curettage might be necessary. Smaller lesions and younger patients may experience spontaneous regression, a factor that is necessary to consider [5].

## Conclusions

Despite advancements in diagnostic techniques and biochemical tests that enable early detection of PHPT, it is essential to keep PHPT in mind as a potential diagnosis when hypercalcemia and radiographic evidence of multiple lytic lesions are observed. This consideration becomes especially important once more common causes such as malignancy have been ruled out. Maintaining a high level of suspicion can lead to a prompt diagnosis and treatment.
